# Gastric T-cell lymphoma associated with hemophagocytic syndrome

**DOI:** 10.1186/1477-7819-2-34

**Published:** 2004-10-19

**Authors:** Rika Fukui, Fumitake Hata, Takahiro Yasoshima, Ryuuichi Denno, Minoru Okazaki, Kiyoshi Kasai, Masaaki Sato, Toshio Homma, Keisuke Ohno, Yoshiyuki Yanai, Katsuya Sogahata, Hidefumi Nishimori, Koichi Hirata

**Affiliations:** 1 First Department of Surgery, Sapporo Medical University School of Medicine, South-1, West-16, Chuo-ku, Sapporo 060-8543, Japan; 2 First Department of Pathology, Sapporo Medical University School of Medicine, South-1, West-16, Chuo-ku, Sapporo 060-8543, Japan; 3 Department of Clinical Pathology, Sapporo Medical University School of Medicine, South-1, West-16, Chuo-ku, Sapporo 060-8543, Japan; 4 Department of Surgery, Shinsapporo Keiaikai Hospital, East-5, Ooyachi higashi, Atsubetsu-ku, Sapporo 004-0041, Japan

## Abstract

**Background:**

Lymphoma-associated hemophagocytic syndrome (LAHS) occurs in mostly extra nodal non-Hodgkin's lymphoma. LAHS arising from gastrointestinal lymphoma has never been reported. Here we report a case of gastric T-cell lymphoma-associated hemophagocytic syndrome.

**Case presentation:**

A 51-year-old woman presented with pain, redness of breasts, fever and hematemesis. Hematological examination revealed anemia. Gastroscopy revealed small bleeding ulcers in the stomach and the computed tomography scan showed liver tumor. She underwent total gastrectomy for gastrointestinal bleeding and the histopathology revealed gastric T-cell lymphoma. She continued to bleed from the anastomosis and died on the 8th postoperative day. Autopsy revealed it to be a LAHS.

**Conclusions:**

If Hemophagocytic syndrome (HPS) occurs in lymphoma of the gastrointestinal tract, bleeding from the primary lesion might be uncontrollable. Early diagnosis and appropriate treatment are needed for long-term survival.

## Background

Hemophagocytic syndrome (HPS) in adults is characterized by reactive and systemic proliferation of benign histiocytes that phagocytose blood cells [1]. It is often associated with infections, malignant neoplasms, autoimmune diseases and various immunodeficiencies. Lymphoma-associated hemophagocytic syndrome (LAHS) mostly occurs from extra nodal lymphoma and is known to have a poor prognosis. Here we report a case of LAHS arising from gastric lymphoma with a fulminant clinical course and difficult diagnosis until the time of autopsy.

## Case presentation

**Figure 1:**
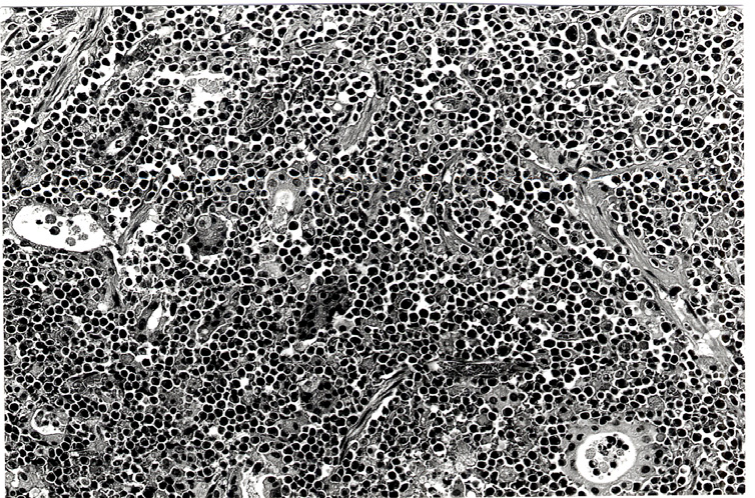
Photomicrograph showing medium-large sized atypical lymphoid cells with pleomorphic features in the stomach suggesting a gastric lymphoma (Hematoxylin and Eosin, ×170).

A 51-year-old female was admitted on May 9, 1995, because of severe hematemesis. The patient had been treated elsewhere for one month for pain and redness of both breasts and fever (≥ 38°C). There was no generalized lymphadenopathy. On gastroscopic examination multiple small ulcers were observed in the stomach. An abdominal computed tomographic (CT) scan showed liver tumor and a normal spleen. Hematological and biochemical examination at admission showed the following results: RBC 352 × 10^4^/mm^3^, hemoglobin 10.3 g/dl (post transfusion), WBC 4,900/mm^3^, Platelets 51,000/mm^3^, serum albumin 1.5 g/dl, total bilirubin 0.6 mg/dl, AST 691 IU/l, ALT 187 IU/l, LDH 2976 IU/l, fibrinogen 134 mg/dl, FDP 10 μg/ml, and AT-III 40%. Bleeding from the stomach continued and did not stop with conservative treatment; therefore, two days later the patient underwent total gastrectomy and a partial liver resection. Histopathology of the resected specimen showed it to be a gastric lymphoma (pleomorphic medium-large cell type, non-Hodgkin's T-cell lymphoma) with liver metastasis (Fig. 1). From first postoperative day (POD), bleeding from the esophagojejunostomy continued; the patient developed disseminated intravascular coagulopathy and died on 8^th ^postoperative day.

**Figure 2:**
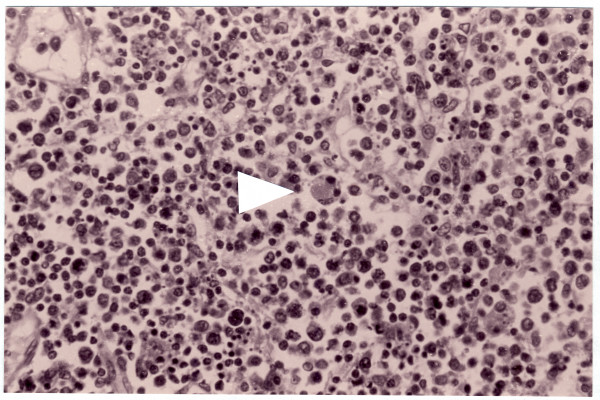
Photomicrograph of the lymph node at autopsy illustrating histiocytes that show hemophagocytosis of normoblast in a lymph node (Hematoxylin and Eosin, ×200).

On autopsy, malignant lymphoid cell infiltration and hemophagocytosis were observed in the liver, spleen, heart, small bowel, lung, both breasts, kidney, pancreas, uterus, and gastroduodenal lymph nodes (Fig. 2). The bone marrow presented hyperplasia and hemophagocytic macrophages but no infiltration by lymphoma cells. Immunohistochemically the neoplastic cells were positive for T-cell marker UCHL1 (CD45RO) and EBV by EBER *in situ *hybridization. The final diagnosis was EBV-related T-cell LAHS.

## Discussion

HPS is a clinicopathological entity characterized by systemic proliferation of benign hemophagocytic histiocytes, fever, cytopenia, liver dysfunction, hepatosplenomegaly, and coagulopathy [1]. This syndrome has been observed during the clinical course of a wide variety of disorders, including viral infections and malignant neoplasms. Diagnostic guidelines of Henter *et al*, [2] are widely used for the diagnosis of HPS. However, these guidelines are not satisfactory in diagnosing HPS in adults; therefore, a number of studies on adult HPS have used their own criteria [1,3,4]. On the other hand for the diagnosis of LAHS, in addition to the clinical features, it is also important to confirm the presence of malignant lymphoid cells histopathologically. Takahashi *et al*, [5] has proposed a set of new diagnostic criteria for adult LAHS that has been detailed in Table 1.

**Table 1:** Diagnostic criteria for adult lymphoma associates hemophagocytic syndrome (LAHS)

1 High fever for more than a week (peak 38.5°C)
2 Anemia (Hb < 9 g/dl) or thrombocytopenia (platelet < 100,000 μ/l)
3 a) LDH ≥ 2 × upper limit
b) Hyperferritinemia (≥ 1,000 ng/dl)
c) Hepatosplenomegaly on CT, US or MRI
d) FDP ≥ 10 μg/ml
4 Hemophagocytosis in bone marrow, spleen or liver
5 No evidence of infection
6 Histopathologically confirmed malignant lymphoma
A diagnosis of LAHS requires that all of the above conditions are fulfilled.
Of the item 3, at least two of the four sub-items (a~d) should be fulfilled.
When item 1 to item 5 are present for 2 weeks and glucocorticoid or γ-globulin therapy is not effective, a diagnosis of probable LAHS can be made and chemotherapy against malignant lymphoma can be started.

In Japan, T-cell LAHS accounts for 48.5% of all adult LAHS [5]. T-cell LAHS mostly occurs in extra nodal, especially nasal, cutaneous, or malignant lymphoma involving liver and spleen. There have been no reports on LAHS from gastric lymphoma. As the diagnosis in the present case was made at autopsy it is not clear as to when the HPS occurred initially. One possibility is the setting of disseminated T-cell lymphoma. This is supported by the patient's fever, which continued for one month, liver dysfunction, and coagulopathy, which existed from the initial stage of the disease, however the bone marrow did not show any lymphoma infiltration. It could also be considered that the hemophagocytic syndrome occurred as a result of the surgery as pancytopenia and hepatosplenomegaly were not observed before the operation and hemophagocytosis was not recognized on histopathological examination in the resected stomach. In T-cell lymphoma, the hemophagocytic syndrome is assumed to be caused by cytokines, especially, tumor necrosis factor-α, and interferon-γ released from neoplastic T-cells [4,6]. Uncontrolled secretion of cytokines may stimulate the proliferation and phagocytic activity of macrophages. It seems likely that hypercytokinemia due to surgical resection might have contribute to the development of HPS in the present case. In our opinion the former is more likely however based on the findings of this case the second hypothesis too cannot be rejected.

The poor prognosis of LAHS, especially T-LAHS, is well known. The median survival time from the diagnosis is reported to be 143 and 69 days respectively in Japan [5]. For LAHS prompt initiation of treatment with multi agent chemotherapy is required to improve the symptoms and survival [7]. Bone marrow transplantation is considered to be a treatment for chemotherapy-resistant LAHS [8]. The median survival time of LAHS patients without chemotherapy is only 11 days [5].

In this case, the initial presentation was mastalgia and hence it took a considerable amount of time to reach a diagnosis. Furthermore, bleeding from the anastomosis continued leading to a rapidly progressive fatal clinical course.

In HPS occurring in lymphoma of the gastrointestinal tract uncontrollable bleeding from the primary lesion might occur. Therefore, an earlier diagnosis of HPS should be made by bone marrow aspirates, and appropriate treatments should be started as soon as possible. Surgery if performed, must be performed with utmost caution.

## Conclusions

LAHS could also occur from lymphoma of the gastrointestinal tract. For long-term survival; early diagnosis and appropriate treatment are needed. Surgery if performed without a proper diagnosis could prove fatal.

## Competing interests

The author(s) declare that they have no competing interests.

## Authors' contributions

**RF, FH, TY, RD, KO and KH **were gastrointestinal surgeons.

**MO **referred this patient to us.

**KK and MS **performed pathological examination and the autopsy.

**KS** was a member of the intensive care team.

**TH, YY, HN **gave us helpful comments about the manuscript
